# Turning WAT into BAT: a review on regulators controlling the browning of white adipocytes

**DOI:** 10.1042/BSR20130046

**Published:** 2013-09-06

**Authors:** Kinyui Alice Lo, Lei Sun

**Affiliations:** *Institute of Medical Biology, 8A Biomedical Grove, #06-06 Immunos, Singapore 138648, Singapore; †Cardiovascular and Metabolic Disorders, Duke-NUS Graduate Medical School, 8 College Road, Singapore 169857, Singapore; ‡Institute of Molecular and Cell Biology, 61 Biopolis Drive, Proteos, Singapore 138673, Singapore

**Keywords:** beige fat, browning, peroxisome-proliferator-activated receptor γ (PPARγ), peroxisome-proliferator-activated receptor γ coactivator 1-α (PGC-1α), PR domain containing 16 (PRDM16), white adipocytes, 4E-BP1, eukaryotic translation initiation factor 4E binding protein 1, ANP, atrial natriuretic peptide, ATF2, activating transcription factor 2, BAT, brown adipose tissue, BMP7, bone morphogenetic protein 7, C/EBP, CCAAT/enhancer-binding protein, Cidea, cell death-inducing DFFA-like effector a, CNP, cardiac natriuretic peptide, COX2, cyclo-oxygenase 2, CtBP, C-terminal-binding protein, EBF2, early B cell factor-2, eIF4E, eukaryotic translation initiation factor 4E, Elovl3, elongation of very long chain fatty acids (FEN1/Elo2, SUR4/Elo3, yeast)-like 3, FGF21, fibroblast growth factor 21, FoxC2, forkhead box protein C2, MAPK, mitogen-activated protein kinase, miRNA, microRNA, Myf5, myogenic factor 5, NPRC, natriuretic peptide receptor C, PGC-1α, peroxisome-proliferator-activated receptor γ coactivator 1α, PGI_2_, prostacyclin, PKA, protein kinase A (=cAMP-dependent protein kinase), PKG, protein kinase G (=cGMP-dependent protein kinase), PPARγ, peroxisome-proliferator-activated receptor gamma, PRDM16, PR domain containing 16, pRb, retinoblastoma protein, SRC-1, steroid receptor coactivator-1, SVF, stromal vascular fraction, TBX15, T-box 15, TFAM, mitochondrial transcription factor A, TIF2, transcriptional intermediary factor-2, TRPV4, transient receptor potential vanilloid 4, UCP1, uncoupling protein 1, WAT, white adipose tissue

## Abstract

Adipose tissue has a central role in the regulation of energy balance and homoeostasis. There are two main types of adipose tissue: WAT (white adipose tissue) and BAT (brown adipose tissue). WAT from certain depots, in response to appropriate stimuli, can undergo a process known as browning where it takes on characteristics of BAT, notably the induction of *UCP1* (uncoupling protein 1) expression and the presence of multilocular lipid droplets and multiple mitochondria. How browning is regulated is an intense topic of investigation as it has the potential to tilt the energy balance from storage to expenditure, a strategy that holds promise to combat the growing epidemic of obesity and metabolic syndrome. This review focuses on the transcriptional regulators as well as various proteins and secreted mediators that have been shown to play a role in browning. Emphasis is on describing how many of these factors exert their effects by regulating the three main transcriptional regulators of classical BAT development, namely PRDM16 (PR domain containing 16), PPARγ (peroxisome proliferator-activated receptor γ) and PGC-1α (peroxisome proliferator-activated receptor γ coactivator 1α), which have been shown to be the key nodes in the regulation of inducible brown fat.

## INTRODUCTION

There are two types of adipose tissue, namely WAT (white adipose tissue) and BAT (brown adipose tissue). The main function of the former is to store excess energy in the form of TAGs (triacylglycerols), whereas the latter is specialized to dissipate energy as heat through UCP1 (uncoupling protein 1)-mediated uncoupling of oxidative phosphorylation from ATP synthesis [1]. The classical BAT and WAT have different developmental origin: brown preadipocytes express skeletal muscle gene signature but their white counterparts do not [2]. Lineage tracing experiments in mice also shows that BAT, similar to skeletal muscle, arises from Myf5 (myogenic factor 5)-expressing precursors [3].

Recently, it was discovered that certain depots of WAT, when subjected to certain stimuli, could take on a BAT phenotype: the browning of WAT. Upon physiological stimuli such as chronic cold exposure, hormonal stimuli such as irisin, pharmacological treatment such as PPARγ (peroxisome proliferator-activated receptor γ) agonist or β-adrenergic stimulation, a brown-fat-like gene expression program [e.g. *UCP1*, *Cidea* (cell death-inducing DFFA-like effector a) and *Dio2* (diodinase 2)] is induced in a subset of Myf5^−^ adipocytes in WAT. These inducible or recruitable brown adipocytes, also known as brite (brown in white) or beige adipocytes, have low thermogenesis activity and a small number of mitochondria at basal state; however, once activated, they possess many biochemical and morphological features of BAT, such as the presence of multilocular lipid droplets and multiple mitochondria. The inducible expression of *UCP1* and other genes related to mitochondrial biogenesis in response to appropriate external cues results in a cell type that is competent in adaptive thermogenesis and energy expenditure similar to classical BAT. Indeed, browning of WAT has been shown to have anti-obesity and antidiabetic effects in rodent models [17].

While beige adipocytes could be perceived as the result of ‘transdifferentiation’ of WAT to BAT in response to appropriate stimuli, a recent study suggests that beige adipocytes are a new type of adipocytes derived from progenitors distinct from WAT and BAT [4]. Wu et al. showed that a subset of precursor cells within murine subcutaneous adipose tissue have a gene expression pattern distinct from both white and classical brown adipocytes in response to forskolin treatment. Furthermore, these cells could give rise to beige cells [4]. Regardless whether browning is simply the result of ‘transdifferentiation’ of WAT to BAT upon external cues or the response of a distinct cell type in WAT that processes an intrinsic propensity to take on a BAT phenotype, the focus of this review is on the known modulators that induce or inhibit the browning process *per se*.

Studies using PET-CT (positron emission tomography-computed tomography) have revealed a high prevalence of metabolically active regions around the supraclavicular areas in adult human subjects, and biopsies from these regions are enriched in UCP1-positive cells [5–8]. The detected BAT activity negatively correlates with BMI (body mass index) and/or body fat [6,9,10]. Markers selective for brown or beige adipocytes identified from rodent studies have provided a tool to investigate the molecular signature of these UCP1-positive cells in human adults [4]. Gene expression signature comparison demonstrates that beige fat markers such as *CD137*, *TMEM26* (transmembrane protein 26) and *TBX1* (T-box 1) are enriched in human ‘brown’ fat samples in comparison with white-fat controls. This strongly argues that these metabolic active UCP1-positive cells could be beige adipocytes [4,11]. However, recent studies suggest that bona fide classical brown adipocytes do exist in human infants [12] and adults [13]. mRNA signature analysis of human adult BAT and isolated preadipocytes differentiated *in vitro* shows signs of classical BAT and beige precursors [14]. These studies highlight that humans, similar to rodents, possess both classical brown fat and beige fat.

Given that there are multiple ways to induce browning in rodents, it is plausible that the browning process could be manipulated to achieve positive therapeutic outcomes in humans [15]. In this review, we will discuss the regulators that have been shown to play important roles in the browning of WAT mainly from mouse studies. The primary focus is on several representative transcription factors and co-regulators that affect browning (Table 1). Also discussed are several hormones, secreted factors and non-transcriptional regulators that are involved in this process (Table 2). Regarding the pharmacological and nutritional agents that promote browning and adaptive thermogenesis in BAT and beige fat, readers can refer to recently published reviews [16,17].

**Table 1:** Transcription regulators involved in the process of browning Listed in Table 1 are the major transcriptional regulators mentioned in the text, transcriptional regulators are listed in alphabetical order.

Regulator	Type*	Model system	Role(s)	Ref
C/EBPα	+, TF	Cultured adipocytes derived from mouse embryonic fibroblasts	Repressing expression of white-fat genes	[25]
EBF2	+, TF	Mouse model, primary SVF cells differentiated to adipocytes	Recruiting PPARγ to BAT genes	[24]
FoxC2	+, TF	Transgenic mice overexpressing FoxC2 in fat	Leading to increase in expression of *UCP1*	[39]
PGC-1α	+, core coregulator	Human subcutaneous fat, PGC1-α knockout mouse	Needed for induction of *UCP*1 and other BAT-specific genes in WAT	[19,36]
PPARγ	+, core TF	Primary adipocytes, mouse model	Full agnoist of PPARγ is needed for browning	[21,23,25]
PRDM16	+, core TF	Ap2-PRDM16 transgenic mouse, primary adipocytes	Needed for induction of browning in subcutanoues fat	[21,23,27,56]
Rb and p107	-, TF	p107 knockout mice, primary adipocytes	Repressing expression of *PGC-1*α	[37]
SIRT1	+, coregulator	Mouse model	Deacetylase PPARγ, leading to its recruitment to PRDM16	[23]
SRC1 and TIF2	-, TF	Whole-body TIF2 knockout mice	WAT from TIF2 knockout mice shows morphology associated with browning	[40]
TBX15	+, TF	Primary cells isolated from wild-type mouse	Needed for BAT and brite fat adipogenesis; upregulating expression of *UCP1* and related genes	[41]
TFAM	-, mitochondrial TF	Adipose-specific knockout mice	Knocking down TFAM increases mitochondrial oxidation capacity due to complex I deficiency and greater uncoupling	[42]
TLE3	-, coregulator	Mouse model, 10T1/2-CAR cell line, 293 cells	Disrupting the interaction of PRDM16 and PPARγ	[30]

*Type indicates whether the regulator has a positive (+) or negative (−) effects on browning and whether the regulator is a transcription factor (TF) or coregulator

**Table 2:** Hormones, secreted proteins and others involved in the process of browning Listed in Table 2 are the major non-transcriptional regulators mentioned in the text.

Regulator	Type	Model system	Role(s)	Ref
4E-BP1	−, others	Mouse model	Negative regulator of PGC-1α protein	[48]
BMP7	+, secreted protein	Brown adipocyte cell line, C3H10T1/2 cell line, BMP7 null mouse	Essential for brown-fat development	[46,47]
CNP	+, hormone	NPRC knockout mice; differentiated human multipotent adipose-derived stem cells	Inducing browning in WAT, acting through PKG	[45]
COX-2	+, others	COX-2 knockout mouse, transgenic mice overexpressing COX-2, C3H10T1/2 cell line, primary adipocytes	Essential for cold- and β-adrenergic-induced browning	[50,51]
FGF21	+, secreted protein	Primary adipocytes, FGF21 knockout mice	Inducing browning in WAT, dependent on PGC-1α	[38,44]
Irisin	+, hormone	Muscle-specific PGC-1α transgenic mice	Inducing browning in WAT, dependent on PPARα	[36]
miR-133	−, micro RNA	Myf5+ brown precursors, Myf5− preadipocytes from subcutaneous WAT	Negatively regulating PRDM16	[53]
miR-193b-365	+, micro RNA	Primary adipocytes	Essential for brown-fat development	[52]
miR-196a	+, micro RNA	Mouse model, primary WAT-pregenitor cells, 3T3-L1	Repressing Hoxc8, de-repressing C/EBPβ	[54]
TRPV4	−, others	3T3-F442A, differentiated primary mouse adipocytes, whole-body TRPV4 knockout mice	Negative regulator of PGC-1α	[49]

*Type indicates whether the regulator has a positive (+) or negative (−) effects on browning and whether the regulator is a hormone, secreted factor, microRNA or others.

## THREE CORE REGULATORS OF BROWNING

### PPARγ

**Figure 1:**
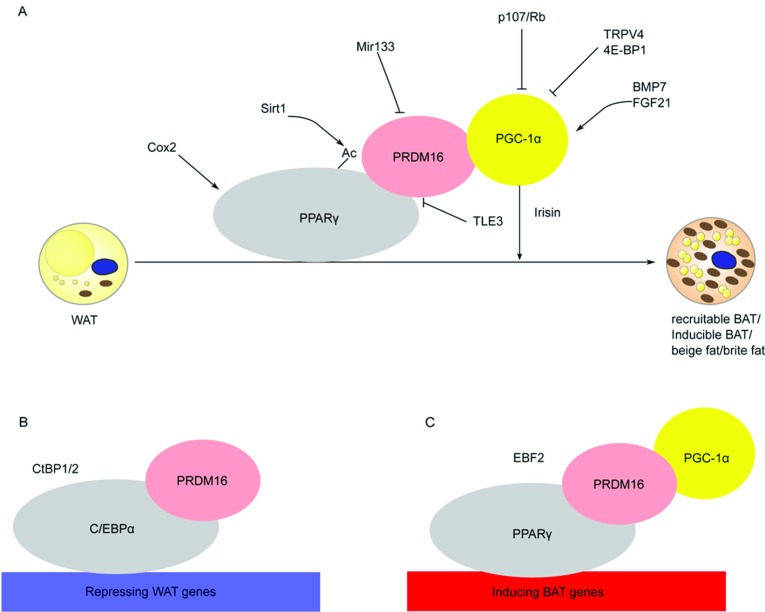
Many different mediators exert their effects on browning through the three core browning transcriptional regulators (**A**) The three core transcriptional regulators of inducible brown fat, namely PRDM16, PPARγ and PGC-1α, are shown as ovals. Other transcriptional regulators are represented in a plain text format. A pointed arrow represents activation, whereas a blunt arrow represents inhibition. See text for detailed explanation of the relationship among the different regulators. Browning involves the repression of white-fat genes (**B**) and the induction of brown-fat genes (**C**) Illustrated is how some of the above-mentioned regulators come together to bring about such effects.

PPARγ is the obligatory transcription factor indispensable for the differentiation and survival of both white and brown adipocytes [18]. Treatment with PPARγ activator rosiglitazone induces the expression of *UCP1*, a BAT hallmark gene responsible for thermogenesis, in WAT of both mouse and human [19,20]. Activation of PPARγ induces brown adipocyte-like cells in mouse epididymally derived white preadipocytes differentiated to mature adipocytes *in vitro* [20], resulting in approximately 10% of the cells being UCP1-positive as revealed by immunocytochemical staining. These cells have increased expression of not only *UCP1*, but also *PGC-1*α (peroxisome proliferator-activated receptor γ coactivator 1α) and other genes related to mitochondrial biogenesis such as *CPT-1M*, *Elovl3* (elongation of very long-chain fatty acids (FEN1/Elo2, SUR4/Elo3, yeast)-like 3) and *Cidea*. These cells are also distinct from classical BAT as they do not express BAT-specific transcription factors *Zic1* (zinc finger protein of the cerebellum 1), *Lhx8* (LIM homeobox 8) and *Meox* (mesenchyme homeobox). They also retain certain WAT-specific gene markers such as *Hoxc9* (homeobox C9), which is not present in classical brown adipocytes. Interestingly, full agonist of PPARγ is required to induce the brown-fat gene programme in subcutaneous WAT in mice, and PRDM16 (PR domain containing 16) is required for this process (Figure 1, and see the PRDM16 section for details) [21].

PPARγ is subjected to various post-translational modifications [22] and functions in tandem with other transcriptional regulators. It was found that SIRT1 (sirtuin 1)-mediated deacetylation of PPARγ is necessary for the recruitment of PRDM16 to the PPARγ transcriptional complex, leading to the process of browning [23]. Through a ChIP (chromatin immunoprecipitation)-Seq experiment comparing genome-wide PPARγ binding sites in primary interscapular BAT and epididymal WAT, EBF2 (early B cell factor-2) was identified as the cofactor that regulates PPARγ binding activity to brown rather than white-fat genes [24]. EBF2 is required for BAT development *in vivo* as demonstrated in late stage embryos from EBF2 knockout mice. When EBF2 is expressed in differentiated SVF (stromal vascular fraction) cells from WAT, PPARγ binding to specific BAT genes increases while that to WAT genes decreases [24].

The browning process not only involves the induction of brown-fat genes but also the repression of white-fat genes (Figure 1). PPARγ was shown to be involved in both the former and the latter: mutation of PPARγ ligand binding site prevents troglitazone-associated inhibition of white-fat genes in 3T3-L1 adipocytes [25]. Such inhibition requires the binding of C/EBPα (CCAAT/enhancer-binding protein α) and CtBP (C-terminal-binding protein) 1 and 2 to the repressed white-fat genes [25]. The interplay of the above-mentioned factors highlights the complexity of the browning process, which involves at least several transcription factors, activators and repressors.

### PRDM16

PRDM16 is the determinant factor of brown-fat development; it is required and sufficient to promote brown adipogenesis in WAT [3,26]. PRDM16 transgenic mice display increased energy expenditure and improved glucose tolerance when fed a high-fat diet. Such improvements are accompanied by a robust development of brown-like adipocytes in subcutaneous, but not epididymal WAT. The cell-autonomous role of PRDM16 in inducing browning in subcutaneous adipocytes was demonstrated: depletion of PRDM16 by shRNA (small hairpin RNA) *in vitro* results in a marked drop in thermogenic gene expression and uncoupled respiration [27]. Similar to PPARγ, PRDM16 is involved in both the induction of BAT genes and the repression of WAT genes. By interacting with CtBP1 and CtBP2 at the promoters of various WAT genes, PRDM16 represses their expression. On the other hand, PRDM16 brings about the induction of BAT-specific genes by associating with the transcriptional coactivators PGC-1α and PGC-1β [28].

It has been shown that certain WAT depots are less prone to browning [29]. For example, visceral/epididymal WAT is less susceptible to browning compared with inguinal/subcutaneous WAT [27]. Browning of primary visceral preadipocytes differentiated to maturity *in vitro* was enhanced by overexpression of PRDM16 [21]. PRDM16 functions synergistically with PPARγ to bring about the browning effect: treatment of primary adipocytes with full agonists of PPARγ leads to the stabilization of PRDM16, while partial agonists, which do not induce browning, have no or only a modest effect on PRDM16 protein stability [21].

In contrast with the BAT-specific role of PRDM16 is the white fat-selective cofactor, TLE3, which promotes lipid storage by blocking the interaction of PRDM16 with PPARγ. Inguinal WAT of adipose tissue-specific TLE3 knockout mice demonstrate browning based on histological analysis; they have increased numbers of cells with multilocular lipid droplets similar to those of beige fat. There is also increase in *UCP1*, *FGF21* (fibroblast growth factor 21) and *Cidea* mRNA expression, although such increase is only observed in inguinal but not epididymal WAT [30].

### PGC-1α

PGC-1α is named after its role as a coactivator of PPARγ. Its expression is elevated upon cold exposure [31]. PGC-1α controls mitochondrial biogenesis and respiration through induction of uncoupling proteins and regulation of nuclear respiratory factors [32]. It is needed for BAT thermogenesis but not differentiation [33]. In primary human subcutaneous white fat, adenovirus-mediated expression of PGC-1α leads to a brown-fat phenotype: there is increased expression of *UCP1*, respiratory chain proteins and fatty acid oxidation enzymes [19]. Adipose PGC-1α deficiency in mice results in a blunted expression of thermogenic and mitochondrial genes in subcutaneous WAT [34]. However, in a different adipose tissue-specific PGC-1α knockout mouse model, PGC-1α was found to be dispensable for the induction of mitochondrial gene expression or biogenesis in WAT, although it is needed for the induction of *UCP1* and other BAT-specific genes in WAT [35]. Although PGC-1α plays an important role in browning, a direct involvement of PGC1-β in this process has not been reported.

In muscle-specific PGC-1α transgenic mice, there is significant up-regulation of *UCP1* and *Cidea* mRNA in the subcutaneous adipose tissue. Immunohistochemistry reveals the presence of UCP1-positive multilocular cells in WAT, a sign of browning [36]. This suggests that there is cross-talk between muscle and fat. Indeed, the browning effects on WAT was at least partly attributed to a muscle-secreted myokine, irisin [36] (see the Irisin section below).

The expression of PGC-1α is regulated by the pRb (retinoblastoma protein) and the Rb family member p107. WAT in p107 knockout mice displays BAT-like features: the presence of many multilocular adipocytes with elevated *PGC-1*α and *UCP1* expression. Interestingly, pRb is markedly down-regulated in p107 knockout mice [37]. As pRb could repress *PGC-1*α expression by binding to its promoter [37], both pRb and p107 are likely to be negative regulators of the browning process because of their effects on PGC-1α. In contrast, FGF21 enhances PGC-1α protein levels in WAT and exerts a positive effect on browning (see the FGF21 section below) [38].

## OTHER TRANSCRIPTION FACTORS AND COREGULATORS

### FoxC2 (forkhead box protein C2)

FoxC2 is a winged helix/forkhead transcription factor that is expressed in both BAT and WAT in mice. In transgenic mice overexpressing FoxC2 under an adipose-specific aP2 (adipocyte fatty acid-binding protein) promoter, there is an increase in the interscapular BAT depot and a reduction in intraabdominal WAT depot. Furthermore, the latter acquired a brown-fat-like histology with regions of small cells harbouring multilocular fat droplets. There is increased expression of *UCP1* and other genes associated with mitochondrial function and biogenesis such as *CoxII* (cytochrome *c* oxidase subunit II) and *PGC-1*α in WAT. One suggested mechanism is that FoxC2 modifies the PKA (protein kinase A (=cAMP-dependent protein kinase)) holoenzyme composition, thus increasing the sensitivity of the β-adrenergic–cAMP–PKA pathway [39].

### SRC-1 (steroid receptor coactivator-1) and TIF2 (transcriptional intermediary factor-2)

Members of the p160 coregulator family, namely TIF2 and SRC-1, have also been shown to play a role in fat storage and thermogenesis [40]. Whole-body TIF2 knockout mice have improved insulin sensitivity and are protected from diet-induced obesity. The BAT of these TIF2 knockout mice has enhanced adaptive thermogenic capacity, whereas their WAT is smaller, accumulates less fat and has decreased expression of genes related to fatty acid uptake and trapping. Although not directly examined, it is plausible that the WAT in TIF2 knockout mice has enhanced potential to browning, given the changes in WAT morphology and the improved metabolic profile of these mice.

### TBX15 (T-box 15)

TBX15 is a homoeodomain transcription factor that plays an important role in development. It is predominantly expressed in classical and inducible brown fat but not WAT [2]. TBX15 is needed for adipogenesis in BAT and inguinal WAT but not epididymal WAT. TBX15 knockdown using siRNA (small interfering RNA) leads to a decrease in both RNA and protein expression of UCP1 and other mitochondrial biogenesis genes [41], the mechanism of which is currently unknown.

### TFAM (mitochondrial transcription factor A)

Besides the various nuclear transcription factors that have been shown to play a role in browning, a mitochondrial transcription factor, TFAM, has also been shown to be important in this process [42]. Adipose tissue-specific TFAM knockout mice are protected from diet-induced obesity, and they have better glucose tolerance through increased mitochondrial oxidation capacity. Although there is no significant difference in UCP1 protein expression between wild-type and the TFAM knockout mice, the latter have increased mitochondrial mass and oxygen consumption in WAT, a hallmark of browning [42]. The above-described changes were attributed to reduced Complex I activity and increased uncoupling (proton leaks) as a result of TFAM knockdown [42].

## SECRETED PROTEINS

### Irisin

Irisin is a recently discovered hormone that is encoded by the gene *Fndc5*. It mediates the beneficial effects of exercise and reduces diet-induced obesity and insulin resistance in mice [36]. Its effects on browning were demonstrated in the subcutaneous WAT in muscle-specific PGC-1α transgenic mice. Such browning effects on white adipocytes can be recapitulated *in vitro* by culturing the cells with media conditioned by PGC-1α-expressing myocytes; this suggests that a secreted factor may be involved. Microarray experiments eventually identified *Fndc5* to be the target of PGC-1α overexpression in muscle, and applying Fndc5 at a concentration of 20 nM to cultured white adipocytes is able to induce a robust browning programme, exemplified by the marked induction of *UCP1* and other BAT genes such as *Elovl3*, *Cox7a* (cytochrome c oxidase subunit VIIa polypeptide 1) and *Otop1* (otopetrin 1). The effects of Fndc5 are mediated at least in part by the transcription factor PPARα as PPARα antagonist significantly reduces the Fndc5-mediated induction of *UCP1* [36].

### FGF21

FGFs are a group of endocrine hormones that are thought to have diverse effects on metabolism [43]. Administering FGF21 *in vivo* and *in vitro* increases the expression of *UCP1* and other brown-fat-related genes in perirenal and inguinal WAT. The effect of FGF21 is dependent on PGC-1α as the induction of brown-fat genes is reduced when FGF21 is administered to primary adipocytes derived from the fat-specific PGC-1α knockout mice [38]. The role of FGF21 in browning was also illustrated in the skeletal muscle-specific autophagy knockout (Atg7^ΔSM^) mice. These mice have reduced muscle and fat mass, increased energy expenditure and improved insulin sensitivity upon high-fat feeding. Interestingly, *UCP1*, *PGC-1*α and the expression of other marker genes of brown-like adipocytes are significantly elevated in perirenal and inguinal WAT of these mice [44]. Microarray analysis reveals that there is marked up-regulation of *Fgf21* gene expression in the skeletal muscle. Such up-regulation was also observed in serum, suggesting a systematic role of FGF21 in browning and energy homoeostasis [44].

### CNP (cardiac natriuretic peptide)

The CNPs, namely the ANP (atrial NP) and the BNP (ventricular NP), are hormones that control fluid and hemodynamic homeostasis. NPs are cleared from the bloodstream through NPRC (natriuretic peptide receptor C). In NPRC knockout mice, NPs are accumulated and NP signalling is enhanced [45]. In these mice, expression of *UCP1*, *PGC-1*α, *CycsI* (cytochrome c somatic I) and *NPRA* (natriuretic peptide receptor) is elevated in BAT, epididymal and inguinal WAT [45]. The white adipocytes also appear smaller, and have less lipid droplets and a deeper reddish-brown tint. The increase in BAT-specific gene expression is also recapitulated in differentiated hMADS (human multipotent adipose-derived stem cells) when ANP is administered, and such responses are additive with β-adrenergic receptor activation. PKG protein kinase G (=cGMP-dependent protein kinase) and PKA are implicated to mediate the downstream effects of NPs and β-adrenergic receptor, respectively, and both signalling pathways converge on p38 MAPK (mitogen-activated protein kinase). ANP induces *UCP1* expression by promoting the binding of PGC-1α and ATF2 (activating transcription factor 2) to the human *UCP1* enhancer regions, and such interactions do not occur when PKG or p38 MAPK activity is inhibited. The effects of NP on the expression of brown-fat markers are also shown in WAT of wild-type mice, demonstrating the browning effects of NPs both *in vitro* and *in vivo* [45].

### BMP7 (bone morphogenetic protein 7)

BMP7 has been shown to play an essential role in brown-fat adipogenesis and regulation of energy expenditure. It triggers the commitment of mesenchymal progenitor C3H10T1/2 cells (a multipotent progenitor cell line) to a brown adipocyte lineage, giving rise to UCP1-positive staining fat pads when such cells are implanted into nude mice [46]. The transcription factor ATF-2 and p38 MAPK seem to mediate the downstream effects of BMP7 as pharmacological inhibition of p38 MAPK blocks BMP7-mediated induction of *UCP1* expression. *UCP1* induction is dependent on PGC-1 as it largely diminishes in brown preadipocytes deficient in both PGC-1α and PGC-1β [46]. Recently, it was demonstrated that constitutive brown fat diminishes as a result of genetic ablation of *Bmpr1a* (type 1A BMP receptor), but a compensatory mechanism comes into action in which browning of WAT occurs. This finding suggests there is potential physiological cross-talk between constitutive and inducible BAT [47].

## OTHERS

### 4E-BP1 [eIF4E (eukaryotic translation initiation factor 4E)-binding protein 1]

4E-BP1, is encoded by the gene *Eif4epb1*. It reversibly binds to eIF4E to repress cap-dependent protein translation. *Eif4ebp1* knockout mice have increased metabolic rate and a reduced WAT mass [48]. Furthermore, the inguinal WAT of these mice exhibits distinctive multilocular appearance of brown adipocytes and increased expression of *UCP1*. The browning effects observed in Eif4ebp1 knockout mice could be due to the increase in PGC-1α translation, though PGC-1α mRNA level is not affected [48].

### TRPV4 (transient receptor potential vanilloid 4)

TRPV4 is a calcium-permeable ion channel. Its role in browning was discovered during a chemical screen in which *TRPV4* was found to be a negative regulator of *PGC-1*α gene expression in 3T3-F442A adipocytes [49]. *TRPV4* inhibition increases the expression of *PGC-1*α, *UCP1* and other mitochondrial genes; such increases are also observed in the subcutaneous and epididymal WAT of whole-body TRPV4 knockout mice. ERK1/2 (extracellular-signal-regulated kinase 1/2) protein kinases are suggested to mediate the gene expression changes. While adipocytes with TRPV4 knockdown exhibit increased respiration rate, TRPV4 knockout mice have increased energy expenditure, reduced inflammation in adipose tissue and are protected from diet-induced obesity [49], attesting to the positive metabolic effects of browning.

### COX (cyclo-oxygenase)-2

COX-2 is the rate-limiting enzyme in the synthesis of prostaglandins. Prolonged adrenergic stimulation with β3-adrenergic agonist induces a BAT-like phenotype, exemplified by the smaller size adipocytes, the presence of multilocular lipid droplets and positive UCP1 staining in WAT of wild-type mice. In contrast, such BAT characteristics are not observed in mice given selective COX-2 inhibitor, or in COX-2 knockout mice [50]. Similarly, cold-induced expression of *UCP1* in inguinal WAT is repressed in mice treated with Cox inhibitor indomethacin and in COX-2 knockout mice [51]. In transgenic mice overexpressing COX-2 (K5COX), excess COX-2 activity leads to browning of intra-abdominal WAT, reduced body weight and fat content despite increased food intake. Chronic COX-2 stimulation also protects mice from diet-induced obesity and dysregulated glucose homoeostasis. It was demonstrated that in an *in vitro* setting, treatment of carbaprostacyclin (a stable analogue of PGI_2_ (prostacyclin), a prostaglandin downstream of COX-2) in C3H10T1/2, mouse adipocyte progenitors isolated from WAT-derived SVF and primary mesenchymal progenitors obtained from human WAT all leads to increased BAT marker gene expression and post-differentiation responsiveness to β-adrenergic stimulation. Such effects are dependent on both the PGI_2_ receptor and PPARγ [50].

### miRNAs (microRNAs)

miRNAs are emerging as new regulators in brown and beige adipocytes. We have previously demonstrated that the *miRNA-193b-365* cluster is strongly enriched in BAT [52]. Inhibition of *miRNA-193b-365* in BAT SVF results in a marked reduction of BAT differentiation. *miRNA-193b-365* works by repressing Runx1t1 (runt-related transcription factor 1; translocated to, 1), an inhibitor of both WAT and BAT adipogenesis, and two pro-myogenic factors, *Cdon* and *Igfbp5* (insulin-like growth factor binding protein 5). Since *miRNA-193b-365* is regulated by PRDM16, potentially through PPARα [52], it is plausible that *miRNA-193b-365* cluster is also involved in the activation of inducible BAT.

PRDM16 is in turn regulated by a miRNA, *miRNA-133*. Upon cold exposure, repression of the transcriptional regulator Mef2 (myocyte enhancer factor 2) leads to decreased expression of *miRNA-133*, which in turn derepresses PRDM16, thereby promoting browning in subcutaneous WAT [53] and skeletal muscle [53]. Another miRNA that is involved in browning both *in vitro* and *in vivo* is *miRNA-196a*. It acts by suppressing the white-fat transcription factor *Hoxc8* (homeobox C8), which in turn relieves the inhibition on *C/EBPβ*, an important regulator of brown adipogenesis [54].

## FUTURE DIRECTIONS AND CONCLUSIONS

Many transcription regulators, proteins and secreted factors have been implicated in the process of browning in WAT. Despite the large number of mediators that have been shown to be involved, it is interesting that a majority of them exert their effects through binding, interacting, activating or inhibiting the main transcriptional regulators of traditional brown-fat development: PRDM16, PPARγ and PGC-1α (Figure 1). More regulators of the above-mentioned key nodes are yet to be discovered.

Certain mouse WAT depots have a greater propensity to form inducible brown fat [29], such as the inguinal WAT and perirenal WAT. On the contrary, epididymal WAT has a lower potential to browning [29]. Such depot differences were also demonstrated in human WAT: human preadipocytes isolated from subcutaneous WAT depot is more prone to browning in response to BMP7 when compared with those isolated from mesenteric or omental WAT [55]. It seems that the higher propensity of subcutaneous WAT to browning is due to the fact that it contains more beige adipocytes/pregenitors than epididymal WAT [4]. However, it remains elusive whether browning is a result of transdifferentiation of certain WAT adipocytes or the activation of beige fat cells that already possess an inherent predisposition to browning. In addition to WAT, certain progenitors residing in skeletal muscle also possess the ability to acquire the BAT characteristics [56]. It is unclear whether these BAT progenitors are different from classical BAT and/or beige fat precursors found in WAT.

The existence of both classical BAT and beige fat in humans present different potential avenues to tackle the growing problems of obesity and metabolic disorders. Given the diverse range of transcription factors, hormones and secreted proteins that could induce browning, it is possible that this process could be manipulated for therapeutic purposes. Various mouse models that have enhanced constitutive or inducible BAT activities are associated with improved metabolic profiles; however, whether browning could have any effects on human energy balance is still unknown. Browning aims to tilt the energy balance towards energy expenditure. Similar endeavours using thyroid hormones or adrenergic agonists have been disappointing, as their systemic effects made them unsuitable for clinical use as treatment of obesity. Before we could harness browning to improve human health, it would be important to understand the mechanism of the browning process in both rodents and humans systemically.
